# Temperature during pregnancy influences the fetal growth and birth size

**DOI:** 10.1186/s41182-016-0041-6

**Published:** 2016-12-14

**Authors:** Harunor Rashid, Miki Kagami, Farzana Ferdous, Enbo Ma, Toru Terao, Taiichi Hayashi, Yukiko Wagatsuma

**Affiliations:** 1 Graduate School of Comprehensive Human Sciences, University of Tsukuba, Tsukuba, Japan; 2 International Centre for Diarrhoeal Diseases Research, Bangladesh (icddr,b), Dhaka, Bangladesh; 3 Department of Clinical Trial and Clinical Epidemiology, Faculty of Medicine, University of Tsukuba, 1-1-1 Tennodai, Tsukuba, Ibaraki 305-8575 Japan; 4 Faculty of Education, Kagawa University, Takamatsu, Kagawa Japan; 5 Center for Southeast Asian Studies, Kyoto University, Kyoto, Japan

**Keywords:** Temperature, Birth length, Birth weight

## Abstract

**Background:**

Birth weight and length have seasonal fluctuations. However, it is uncertain which meteorological element has an effect on birth outcomes and which timing of pregnancy would explain such effect. Therefore, the purpose of this study was to examine temperature effects during pregnancy and which timing of pregnancy has effects on size at birth.

**Methods:**

A large, randomized, controlled trial of food and micronutrient supplementation for pregnant women was conducted in Matlab, Bangladesh (MINIMat Study), where women were enrolled from November 2001 to October 2003. The fetal growth data which included the size at birth and information of their mothers were obtained (*n* = 3267). Meteorological data such as temperature, precipitation, relative humidity, and daily sunshine hours during pregnancy were observed at the nearest observatory site of Bangladesh Meteorological Department.

**Results:**

Infants born in colder months (November–January) were shorter than those born in hot and dry, and monsoon months (mean (SD) of birth length was 47.5 cm (2.2) vs. 47.8 cm (2.1) vs. 47.9 cm (2.1) respectively; *P* < 0.001). Increased temperature during the last month of pregnancy was significantly related with increased birth length with adjustment for gestational weeks and the season at birth, and remained significant with further adjustments for precipitation, sex of infants, maternal early-pregnancy BMI, parity, and education status of the mother (*P* < 0.01). On the other hand, increased temperature at mid-gestation was associated with increased birth weight (*P* < 0.05).

**Conclusions:**

These findings suggest that temperature affects both birth weight and length. The more temperature increased at the last month of pregnancy, birth length became longer. For birth weight, the temperature at mid-pregnancy affected in a positive way.

**Electronic supplementary material:**

The online version of this article (doi:10.1186/s41182-016-0041-6) contains supplementary material, which is available to authorized users.

## Background

Climate change has become a public health priority due to a huge amount of evidence on global warming and the impact of human activities [1]. The changes of average temperature, extreme weather events, such as drought and heavy precipitation, and the duration of seasons also affect the geographic distribution and intensity of transmission of infectious diseases [2–4]. People in developing countries, particularly in tropical areas, are likely to suffer most from climate change due to poverty, poor sanitation, poor health status, high population density, poor health care systems, and political instability that are influenced by the limited ability of health care systems to respond to an increase in the burden of climate-sensitive outcomes [5].

A well-nourished mother is crucial for proper fetus development and adequate birth outcome [6]. The development of fetus also depends on multiple genetic factors and environmental exposure derived from the mother, such as poor nutritional status, poor living environment, and infectious diseases [7]. Therefore, they will be vulnerable to the environmental effects of climate change [8, 9]. Increasing heat events from climate change could reduce 1.7–1.8 g birth weight which could vary by socioeconomic strata as revealed by a recent study in the USA [9]. Physical activity (paid work or work in the household) until shortly before the delivery is a common phenomenon in low- and middle-income countries [10], which might lead to increased vulnerability for changes in external temperature that sometimes result in serious changes in core body temperature [11]. Changes of core body temperature can influence metabolic changes in different nutrients of pregnant women, and that can affect fetal development and growth [12–14].

Several studies found the seasonal variations on birth outcome, and some of those have also examined the association between birth size and meteorological factors [15–18]. Effect of higher and lower outdoor temperature during different stages of pregnancy showed reduced offspring birth weight [19]. An Israeli study demonstrated that increased exposure to sunlight in the last weeks of pregnancy was possibly associated with increased birth weight [18]. Another study suggested the relationship between low birth weight (LBW) and the exposure to low winter temperature during mid-gestation [16].

LBW is a major determinant of mortality and morbidity in neonates, infancy, and childhood. The incidence of LBW in the world is 15%, most of which are born in developing countries [20]. Yasmin et al. reported that more than 60% of LBW infants in Bangladesh were delivered at term [21]. Villar and Belizán found a significant association between the total LBW incidence and the incidence of LBW who was born after 37 weeks of gestation from the data of 25 developing areas [22]. LBW infants who were born after 37 weeks of gestation are thought that they suffered from intrauterine growth retardation (IUGR). However, it has not been assured which meteorological element has an effect upon the birth size and also which timing of such effect during pregnancy would explain the difference in size at birth. Moreover, it is not determined whether temperature exposures during different stages of gestation importantly influence birth weight [19]. Thus, the aim of this study was to examine whether the temperature during pregnancy has an influence on fetal growth and thus on the size of birth.

## Methods

### Study area and population

Matlab is located about 50 km southeast of Dhaka, the capital city of Bangladesh. The area is a typical rural and riverine delta area of Bangladesh. The most common livelihoods are rice cultivation and fishing, and around 85% of the population is Muslim. The International Center for Diarrhoeal Disease Research, Bangladesh (icddr,b) has been maintaining a health and demographic surveillance system in Matlab since 1966. icddr,b and collaborative partners have been conducting randomized trials aiming to improve maternal nutrition through food and micronutrient supplementation in this area since 2001, the Maternal and Infant Nutrition Intervention Study at Matlab (MINIMat study) [23].

In brief, the MINIMat study consists of three combined intervention trials in a group of undernourished pregnant women of rural Bangladesh. All women who were identified as pregnant in the ongoing demographic surveillance system area of icddr,b were recruited for the study. Pregnant women were identified within 6–8 weeks of conception through monthly home visits, and their pregnancies were confirmed by two times of urine tests and ultrasonography. Pregnant women were enrolled in a 2-year period from November 2001 to October 2003. A total of 5880 women were identified as eligible for the study. Of these women, 1444 were excluded because 504 refused to participate. A further 604 women had to be excluded due to having a fetus whose GA >14 weeks exceeded the limits for the study and 217 no longer having a viable fetus by ultrasound, 112 migrated out from the study area, and 8 excluded for other reasons. Of the 5580 pregnant women, 4436 women were successfully enrolled in the MINIMat study. Among infants of these women, birth weight of 3298 infants was measured. Of these infants, the number of singleton birth was 3267 born from May 2002 to June 2004 and were considered as the current study population. In the present study, the mean person-years of follow-up was 0.71 year or 8.6 months.

### Information of mothers and newborns

The information for mothers contained maternal weight, height, age, last menstrual period (LMP) by ultrasonography, formal education years, and parity. Maternal weight and height were measured at enrollment, and other information was collected during home visits. The study data contained the information of infants such as birth weight, birth length, sex, date of birth, and gestational week at birth. Birth weight was measured by using beam scales (Seca GmbH, UK) that were precise to 10 g. Birth length was measured to the nearest 0.1 cm with locally constructed wooden length board. These measurements were taken within 72 h after birth by study staff. The birth weight and birth length were adjusted by the timing of the measurements after birth [23].

### Meteorological data

The information of meteorological elements was observed at the nearest observatory station in Chandpur, located about 10 km from the central Matlab, by the Bangladesh Meteorological Department. The meteorological data between January 2001 and December 2004 was obtained.

The mean daily temperature, precipitation, and relative humidity were calculated from the data collected every 3 h. Ten time points during pregnancy were selected (8, 12, 19, 24, 28, and 30 gestational weeks; 6, 4, and 2 weeks before birth; and at birth). The MINIMat study was scheduled for enrollment at 8–10 weeks and three subsequent clinic visits at 14, 19, and 30 weeks of gestation. However, pregnant women came for ultrasound examinations at slightly varied timings. To have maximum participation, the study allowed to examine the women any time before the next examination schedule. Apart from the fixed timings (8, 14, 19, and 30 weeks), the effect of temperature was tested to have additional time points of two points in each pregnancy trimester between 8 and 30 weeks, i.e., 8 and 12 weeks in the first trimester, 19 and 24 weeks for the second trimester, and 28 and 30 weeks for the third trimester. Close to births, test points were considered in 2-week intervals up to 6 weeks from the birth point (before 6, 4, and 2 weeks and at birth), since 7% of the children were preterm born and other children also vary for birth timing (varied for gestation weeks at birth). The 2-week moving average of each meteorological element was calculated for each point from daily average data. For example, average temperature at 8 weeks of gestational age was calculated by using moving average of temperature at 7 and 8 weeks (up to the end of weeks) of gestation. Moving averages for other points were also calculated in the same manner except the point at birth. As for the temperature at birth, the 2-week moving average before the birth was used. Fetus development is a steady growth process. To observe slight effect of temperature, we need an incubation period to assess the effect of temperature on fetal growth and birth outcome. However, we have also done sensitivity analyses with 1-week moving average, which showed a smaller effect size compared to the 2-week moving average (Additional file 1: Table S1 and Additional file 2: Table S2). An earlier study considered the 2-week lag of temperature on mortality analysis; however, their objective was different from the present study [4]. In Bangladesh, three broad seasons are generally observed [24]; thus, the present study also classified seasonality in three groups as follows: the winter (October to February), the hot and dry season (March to May), and the monsoon season (June to September).

### Statistical analysis

Mean differences of temperature and precipitation by month were examined by one-way ANOVA. The correlation between birth weight or length and meteorological elements were assessed by Pearson’s correlation coefficient. The correlation of possible confounders such as maternal education level and mother’s body mass index (BMI) at early-pregnancy was also examined. Seasonal and monthly differences of birth weight or length were examined by one-way ANOVA. Simple regression analyses for birth weight or length and temperature or precipitation at each gestation point were conducted. Multivariable regression analyses for these associations were conducted in separate models for each gestation points with adjustments for gestational week at birth, season at birth, sex of infant, parity, early-pregnancy BMI, and education level of mothers, and further with precipitation at the same gestation week. Gestation week at birth, parity, early-pregnancy BMI, and precipitation were incorporated as continuous variables as they were distributed normally. Infant sex, season at birth (three seasons), and mother’s education years (0, 1–6, ≥7) were categorical variables in the multivariable models.

For examining non-linear association, the study tested by incorporating polynomial terms of the temperature into the models. The study checked the quadratic models of the temperature and birth weight and birth length, and then found that beta coefficients of temperature did not significantly appear as compared to those in linear regression models. Therefore, the study used linear models.

Stratified analyses of the mean temperature at each gestation point for birth weight or length were given by maternal early-pregnancy BMI (<18.5 or ≥18.5 kg/m^2^), as nearly one third of the study participants were undernourished (BMI <18.5). *P* value of <0.05 at two-sided test was considered as statistically significant. IBM SPSS (version 23; New York, USA) was used for statistical analyses.

## Results

### Characteristics of mothers and newborns

Table 1 shows characteristics of 3267 mothers and their singleton newborns. The mean (range) of maternal age was 26.5 (14–50) years old. Approximately, 41% of mothers had more than 7 years of education. There were 918 women (28.2%) whose BMI at early pregnancy was less than 18.5 kg/m^2^. Mothers with less formal education years showed lower early pregnancy BMI (32, 30, and 24% in 0, 1–6, and ≥7 years of education, respectively). Of infants, 1663 (51%) were male. The mean (range) birth weight was 2693 (1059–4249) g. About 30.5% of infants were born with low birth weight (LBW); however, 80% of those infants had full-term delivery (born after 37 weeks of gestation). The number of winter newborn was 1564 (48%), one-third of infants were born in the monsoon season, and the rest of the infants were born in the hot and dry season.

**Table 1:** Characteristics of mothers and newborns (*n* = 3267)

	Number	Percent
Characteristics of mothers		
Age in years, *n* = 3264		
<20	486	14.9
20 to <30	1868	57.2
≥30	910	27.9
Early-pregnancy BMI (kg/m^2^), *n* = 3257		
<18.5	916	28.0
18.5 to <25.0	2151	65.9
≥25.0	200	6.1
Education years		
0	1056	32.3
1~6	874	26.8
≥7	1337	40.9
Parity, *n* = 3252		
0	1030	31.7
1	914	28.1
2	699	21.5
3	358	11.0
≥4	251	7.7
Characteristics of newborns		
Male	1663	50.9
Birth weight (<2500 g)	997	30.5
Birth length (<47 cm), *n* = 3256	999	30.7
Gestation week at birth <37	259	7.9
Season at birth		
Winter (October to February)	1564	47.9
Hot and dry (March to May)	694	21.2
Monsoon (June to September)	1009	30.9

### Seasons and birth size

**Fig. 1:**
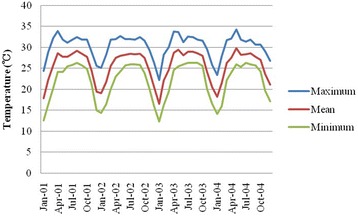
Seasonal variation of maximum, minimum, and mean temperature from 2001 to 2004

The means of temperature, precipitation, and birth size among seasons are shown in Table 2. During the monsoon season, the precipitation was almost eight times higher than the precipitation in winter. The pattern of the mean temperature was similar to those of maximum temperature and minimum temperature (Fig. 1).

**Table 2:** Mean and standard deviation of temperature, precipitation, and birth size by season

Season	Temperature (°C)	Precipitation (mm)	Birth weight (g)	Birth length (cm)*
Winter (October to February)	22.4 ± 3.8	1.5 ± 9.0	2689.7 ± 411.3	47.5 ± 2.2
Hot and dry (March to May)	27.4 ± 2.4	3.6 ± 11.6	2724.4 ± 412.7	47.8 ± 2.1
Monsoon (June to September)	28.5 ± 1.1	12.8 ± 23.1	2688.1 ± 409.1	47.9 ± 2.1

*P < 0.001

The mean birth weight for males was heavier than that for females (2764.0 vs. 2687.7 g; *P* = 0.001). Infants who were born in hot and dry season tended to be heavier; however, it was not statistically different (*P* > 0.05). A seasonal pattern of birth length was seen. Infants who were born in winter tended to be shorter than infants who were born in the other two seasons (47.5 cm in winter, 47.8 cm in hot and dry, 47.9 cm in monsoon; *P* < 0.001) (Table 2).

### Temperature at gestation weeks and birth weight

Birth weight and birth length were strongly correlated (*r* = 0.779; *P* < 0.001). Birth weight was also correlated with gestation week at birth (*r* = 0.475; *P* < 0.001), parity (*r* = 0.108; *P* < 0.001), education level (*r* = 0.101; *P* < 0.001), and early-pregnancy BMI (*r* = 0.146; *P* < 0.001).

Results of liner regression analyses for birth weight are shown in Table 3. The temperature at 28 and 30 weeks of gestation and 6 weeks before birth was negatively associated with birth weight (*P* < 0.05). After adjustment for gestational week and season at birth, the temperature at 12 to 19 weeks of gestation had significant positive associations with birth weight (*P* < 0.05). When did further adjustment for sex of infant, early-pregnancy BMI, mother’s education, and parity, the significant association with birth weight was only seen in the temperature at 19 weeks of gestation. After adding the precipitation for adjustment, as temperature (1 °C) increased at 19 weeks of gestation, birth weight was increased up to 4.7 g.

**Table 3:** Regression coefficients for the association between birth weight and temperature at different gestational points (*n* = 3267)

Week	Univariate		Multivariate 1*		Multivariate 2**		Multivariate 3***	
	*β*	*P* value	*β*	*P* value	*β*	*P* value	*β*	*P* value
8	−1.176	0.537	−5.273	0.072	−4.101	0.141	−3.824	0.180
12	1.324	0.504	6.864	0.033	5.334	0.081	5.970	0.052
19	0.545	0.786	5.113	0.023	4.226	0.048	4.733	0.028
24	−2.804	0.151	3.155	0.308	2.364	0.421	1.849	0.541
28	−3.82	0.049	−0.688	0.809	−0.803	0.766	−1.431	0.613
30	−4.156	0.028	−0.596	0.801	−0.343	0.878	−0.973	0.679
Before birth								
6	−4.236	0.022	−1.279	0.495	−1.045	0.557	−1.772	0.342
4	−2.326	0.205	0.402	0.830	0.398	0.823	0.530	0.775
2	−0.102	0.956	2.845	0.160	2.264	0.239	3.080	0.123
At birth	0.595	0.747	4.027	0.61	3.229	0.113	3.567	0.860

*Adjusted for gestational week at birth and season

**Adjusted for sex of infant, BMI, mother’s education, parity, season at birth, and gestational week at birth

**Adjusted for precipitation, sex of infant, BMI, mother’s education, parity, season at birth, and gestational week at birth

Table 4 shows coefficients of stratified analyses adjusted for gestational week and season at birth. In mothers whose BMI was less than 18.5 kg/m^2^, the low temperature during the last month of pregnancy was associated with the lower birth weight (*P* < 0.05) after adjustment for sex of infant, early-pregnancy BMI, mother’s education level, parity, and precipitation. The temperature at 6 weeks before birth was associated with both groups (*P* < 0.05). When the temperature at 6 weeks before birth was increased, birth weight was increased in infant whose mother had lower BMI, while it was decreased in infant whose mother had higher BMI.

**Table 4:** Regression coefficients for association between birth weight and temperature with stratification of mother’s BMI with adjustments (*n* = 3267)

Week	Multivariate 1*				Multivariate 2**			
	BMI <18.5		BMI ≥18.5		BMI <18.5		BMI ≥18.5	
	*β*	*P* value	*β*	*P* value	*β*	*P* value	*β*	*P* value
8	−4.076	0.347	−0.693	0.802	−6.761	0.192	−3.012	0.388
12	6.357	0.197	5.915	0.090	5.190	0.324	8.878	0.020
17	8.626	0.024	2.501	0.358	7.279	0.053	3.844	0.148
19	5.466	0.127	2.119	0.393	6.971	0.067	4.345	0.101
24	2.414	0.450	−0.904	0.665	9.649	0.078	−0.772	0.835
28	1.344	0.675	−1.755	0.381	7.352	0.173	−5.336	0.115
30	3.224	0.311	−2.228	0.257	8.083	0.072	−4.482	0.111
Before birth								
6	6.484	0.053	−3.556	0.082	7.263	0.041	−5.047	0.024
4	8.100	0.025	−2.021	0.353	7.916	0.026	−1.380	0.533
2	8.891	0.022	0.334	0.889	9.270	0.014	1.003	0.675
At birth	7.219	0.075	1.659	0.507	7.455	0.061	2.614	0.291

*Adjusted for gestational week at birth and season at birth

**Adjusted for gestation week at birth, season at birth, sex of newborn, education level, parity, and precipitation

### Temperature at gestation weeks and birth length

Birth length was correlated with gestation week at birth (*r* = 0.518; *P* < 0.001), parity (*r* = 0.075; *P* < 0.001), education level (*r* = 0.095; *P* < 0.001), and early-pregnancy BMI (*r* = 0.094; *P* < 0.001). Birth length was also significantly correlated with the mean of overall temperature (*r* = 0.090; *P* < 0.001), and precipitation (*r* = 0.045; *P* < 0.01).

Table 5 shows coefficients of multivariate analyses for birth length adjusted for gestational week and season at birth. When the temperature at 8 weeks of gestation increased, birth length became shorter. On the other hand, temperature at the last month of pregnancy affected birth length in a reversed direction. Even after controlling the precipitation, similar results were observed.

**Table 5:** Regression coefficients for the association between birth length and temperature at different gestational points (*n* = 3267)

Week	Univariate		Multivariate 1*		Multivariate 2**		Multivariate 3***	
	*β*	*P* value	*β*	*P* value	*β*	*P* value	*β*	*P* value
8	−0.049	<0.001	−0.051	0.001	−0.045	0.002	−0.041	0.005
12	−0.044	<0.001	−0.001	0.937	−0.003	0.842	0.002	0.903
19	−0.032	0.002	0.008	0.513	0.005	0.632	0.003	0.801
24	−0.024	0.022	0.007	0.653	0.004	0.815	−0.010	0.544
28	−0.008	0.454	0.018	0.234	0.015	0.295	0.004	0.804
30	−0.001	0.918	0.020	0.103	0.019	0.113	0.011	0.382
Before birth								
6	0.012	0.209	0.019	0.054	0.019	0.045	0.012	0.200
4	0.031	0.002	0.032	0.001	0.032	0.001	0.026	0.007
2	0.044	<0.001	0.046	<0.001	0.042	<0.001	0.042	<0.001
At birth	0.050	<0.001	0.056	<0.001	0.051	<0.001	0.051	<0.001

*Adjusted for gestational week at birth and season at birth

**Adjusted for sex of infant, BMI, mother’s education, parity, season at birth, and gestational week at birth

***Adjusted for precipitation, sex of infant, BMI, mother’s education, parity, season at birth, and gestational week at birth

Multicollinearity was examined and variance inflation factor (VIF) for all the models of birth length and weight were less than 2, indicating no serious collinearity impact. Supplementary tables were provided with VIF for models of birth lengths for 6, 4, and 2 weeks before birth and at birth (Additional file 3: Table S3a, S3b, S3c, S3d).

The newborns whose mother’s BMI was less than 18.5 kg/m^2^ were shorter than those mother’s BMI was more than 18.5 kg/m^2^ (47.4 cm (2.2) vs. 47.8 cm (2.2); *P* < 0.001). By stratified analyses with adjusted for gestation weeks and season at birth, temperature of the last month of gestation was significantly associated with birth length for both groups (*P* < 0.05). After adjusted for the sex of infant, early-pregnancy BMI, education level, parity, and precipitation on previous model, correlations were almost unchanged (Table 6). Birth length was associated with the temperature at 8 weeks of gestation for both groups (*P* < 0.05). When the temperature increased at 8 weeks of gestation, birth length was decreased.

**Table 6:** Regression coefficients for association between birth length and temperature with stratification of mother’s BMI with adjustments (*n* = 3267)

Week	Multivariate 1**				Multivariate 2**			
	BMI <18.5		BMI ≥18.5		BMI <18.5		BMI ≥18.5	
	*β*	*P* value	*β*	*P* value	*β*	*P* value	*β*	*P* value
8	−0.042	0.062	−0.025	0.080	−0.060	0.027	−0.038	0.033
12	−0.006	0.814	0.001	0.937	−0.002	0.948	0.011	0.565
17	0.011	0.570	0.002	0.892	0.014	0.482	0.002	0.868
19	0.014	0.456	0.001	0.968	0.023	0.253	−0.002	0.903
24	0.011	0.495	−0.002	0.873	0.047	0.096	−0.030	0.116
28	0.015	0.366	0.004	0.713	0.049	0.079	−0.015	0.396
30	0.020	0.228	0.007	0.462	0.037	0.112	0.001	0.960
Before birth								
6	0.047	0.008	0.007	0.486	0.054	0.003	−0.003	0.793
4	0.064	0.001	0.022	0.054	0.055	0.003	0.018	0.109
2	0.067	0.001	0.036	0.004	0.059	0.003	0.036	0.004
At birth	0.062	0.003	0.046	<0.001	0.057	0.005	0.050	<0.001

*Adjusted for gestational week and season at birth

**Adjusted for gestation week at birth, season at birth, sex of newborn, education level, parity, and precipitation

## Discussion

In this cohort study, we have observed associations between birth size and temperature at various timings of pregnancy, which was supported by previous study findings [15–18]. Present study showed that as temperature increased at the last month of pregnancy, birth length increased. This finding remained unchanged even after the adjustments with precipitation. The temperature of the last month of pregnancy was directly associated with birth length for neonates of both malnourished and well-nourished women. Moreover, lower temperature at the last month of pregnancy was associated with decreased birth weight, particularly for malnourished women. Another important finding of this study was that high temperature exposure in the early trimester at 8 weeks of pregnancy has adverse effects on birth length. To our knowledge, this is the first study to report the effect of temperature for different timings of gestation on birth length.

Exposure in bright sunlight during pregnancy identified as a significant determinant of height [25], and this study outcome partially supported this notion that as daily temperature increased in the last trimester of pregnancy, the birth length increases. Pregnancy health is strongly associated with serum 25-hydroxy vitamin D [26], and the role of vitamin D in bone mineralization is well known [26, 27]. A study in London reported that maternal vitamin D insufficiency at pregnancy as early as 19 weeks can influence fetal femoral development, and suggested that maternal vitamin D should be improved in early pregnancy [14]. The seasonal variation of 25-hydroxy vitamin D concentration was high in summer [28, 29], and the sunlight exposure in summer provided 80% of total annual intake of vitamin D [28]. Thus, if the third trimester is in spring or in summer, the status of maternal vitamin D would be better than in other seasons. An Australian study demonstrated that infants whose mother was vitamin D deficient (<28 nmol/l) at the third trimester had shorter knee-heel length [29]. Another study in Perth, Australia, revealed that higher temperatures constant over pregnancy may adversely affect fetal growth, which was associated with a 2% increase in risk of growth restriction. There was also an indication that higher temperatures in the third trimester predicted declines in fetal growth. These results suggest that heat effects throughout and potentially late in pregnancy may constrain fetal growth independently of ambient air pollutants and other risk factors, particularly in areas systematically subjected to high seasonal peaks [30]. Bangladesh is a tropical country and daily sunlight exposure is a common phenomenon for rural women due to their daily life activities, with a peak in hot and dry season. Therefore, the positive association between high temperature at the third trimester and increased birth length in this study might be partly explained by vitamin D status of mothers [31]. This study, however, did not aim to examine sunshine effect on birth size. Further studies are required to clarify this association.

Present study also found increasing temperature at 6 weeks before birth is associated with reduced birth weight particularly for children of well-nourished women and improved birth weight of children of undernourished women, which has been supported by previous study [19]. That study found a positive association in lower ambient outdoor temperature in the last trimester of pregnancy and reduced birth weight; however, that study did not consider nutritional status of pregnant women. It should be noted that present study included overweight women in the BMI 18.5 and above group. A possible physiological mechanism is that increased temperature lowers body fatness among the overweight women, changes core body temperature, and reduced pregnancy weight gain which leads to reduced birth weight [32]. Association between lower birth weight and low winter temperature during mid-gestation was reported in Northern Ireland, and the present study also found a similar association [16]. Northern Ireland and Bangladesh are located in different geoclimate areas. Therefore, it might be difficult to explain only by the effects of temperature since meteorological elements are correlated with each other. In Bangladesh, flood often happens in monsoon season, and it has powerful influences on human life through the epidemics of diarrhea. After adjusting for precipitation in multivariate analyses, coefficients of temperature at mid-gestation with birth weight were still significant. This could explain the temperature effect on birth size. However, we should be careful to interpret the results from different climate areas in the world. For instance, a winter season could be wet winter or dry winter in different areas.

Increased temperature at birth was associated with adverse effect on birth weight observed in a Greek study [33]. This association was not found in the present study. However, their study aim was to examine the seasonal effect on fetal development, not the temperature effect on it. Therefore, they used only the mean temperature during the month of birth. Moreover, they did not seem to examine other meteorological elements. Further studies are required to consider other meteorological factors.

The placental weight in the second trimester was a strongly significant predictor of birth weight [34]. An animal study reported that heat stress in early pregnancy is associated with reduced placental weight [32]. Another animal study reported that the total placental weight was reduced in ewes which had been exposed to 30 °C for 15 h and 40 °C for 9 h [8]. The ewes were exposed to high temperature. The result suggests that the placental size may be changed by temperature. Higher temperature at the second trimester might relate to heavier placental weight of Bangladeshi pregnant women, and it can be varied by nutritional status, socioeconomic factors, and other related factors. Further studies are required to confirm the relationship between placental weight and temperature during pregnancy. Another possible explanation might be the effect of temperature on growth hormone secretion during pregnancy. Exposure to sunlight during the first trimester of pregnancy stimulates growth hormone release by inhibiting production of maternal pineal melatonin which may effect on birth size and final adult stature [25]. An animal study reported that hormone concentrations of maternal and fetal origin were altered by environment, and heat stress at pregnancy altered endocrine dynamics which reduced birth weight [35]. Therefore, production and secretion of growth hormone or growth hormone releasing factors may interact with atmospheric conditions during pregnancy [35, 36].

Malnourished mothers are more likely to give birth to LBW, IUGR, and preterm infants, as seasonality and direct heat exposure are also associated with the same adverse birth outcome [17, 19]. Therefore, it is clear that exposure to high temperate might have some adverse effects on undernourished women and their growing fetus. However, time of pregnancy trimester with the change of season might influence this effect. The present study could be useful to improve fetal development and reduce the prevalence of LBW since many Bangladeshi fetuses have a problem of the IUGR. Moreover, this study showed that the difference of the temperature effect on size at birth depend on the mother’s early pregnancy nutritional status (BMI). Due to climate change, the mean temperature has been rising globally [37]. It tends to be considered visible or greater effects of temperature on environment and human. However, even a small effect could be greater when it accumulates. Absolute effect of temperature on size is small, but possibly continues until adulthood [38, 39].

One of the limitations of this study was that factors which could affect birth size such as maternal dietary information and maternal vitamin D status during pregnancy were not available. Mothers who participated in the MINIMat study were randomized into six supplementation groups to receive different nutrition supplements during pregnancy. Nevertheless, the effect on birth weight and birth length by supplementation groups was not observed in the MINIMat study [40]; consequently, the present study did not make an adjustment of this supplementation. Another limitation of this study was that the data were only from live-born infants. Stillbirth infants and fetuses who were miscarried were not analyzed in this study. They might have been more affected by temperature because of their vulnerability. Moreover, this is a study of single rural area to assess the temporal temperature variation on birth outcome. The results of this study may be referred only to this study population because of the differences of geoclimate and other factors. Strengths of our study include the fact that we examined temperature effect on several points of pregnancy and on birth outcome. The main strength of our study is the large number of women who participated. A further strength was that this is a single cohort study from developing countries that observed the temperature effect with the consideration of seasonality and precipitation on birth size. These study results may be helpful for other developing countries where fetal growth restriction is prevalent.

## Conclusions

The present study found the significant effect of temperature during pregnancy on birth size. As temperature increased at the last month of pregnancy, birth length was longer. However, the effect varies by the mother’s nutritional status. The present study suggests that pregnant women, particularly whose BMI is less than 18.5, should avoid colder temperatures in late pregnancy. Several other aspects are associated with fetal growth, but it could be reasonable for pregnant women to avoid cold temperature during mid and late pregnancy.

## Additional files

Additional file 1: Table S1. Regression coefficients for the association between birth length and temperature at different gestational points (using 1-week moving average temperature; *n* = 3267). (DOC 34 kb)
Additional file 2: Table S2. Regression coefficients for the association between birth weight and temperature at different gestational points (using 1-week moving average temperature; *n* = 3267). (DOC 1478 kb)
Additional file 3: Table S3. a. Variance inflation factor (VIF) of birth length at 6 weeks before birth. b. Variance inflation factors (VIF) of birth length at 4 weeks before birth. c. Variance inflation factors (VIF) of birth length at 2 weeks before birth. d. Variance inflation factors (VIF) of birth length at birth. (DOC 1119 kb)

## Abbreviations

BMI: Body mass index
GA: Gestational age
icddr,b: International Centre for Diarrhoeal Disease Research, Bangladesh
IUGR: Intrauterine growth retardation
LBW: Low birth weight
LMP: Last menstrual period
MINIMat: Maternal and Infant Nutrition Intervention Study in Matlab

## References

[1] Climate change and human health—World Health Organization. In: McMichael AJ, Campbell-Lendrum DH, Corvalán CF, et al., eds, 2003. http://www.who.int/globalchange/publications/climchange.pdf.

[2] Holy M, Schmidt G, Schroder W (2011). Potential malaria outbreak in Germany due to climate warming: risk modelling based on temperature measurements and regional climate models. Environ Sci Pollut Res Int.

[3] Ebi KL, Helmer M, Vainio J (2008). The health impacts of climate change: getting started on a new theme. Prehosp Disaster Med.

[4] Hashizume M, Wagatsuma Y, Hayashi T, Saha SK, Streatfield K, Yunus M (2009). The effect of temperature on mortality in rural Bangladesh—a population-based time-series study. Int J Epidemiol.

[5] Rylander C, Odland JO, Sandanger TM (2013). Climate change and the potential effects on maternal and pregnancy outcomes: an assessment of the most vulnerable—the mother, fetus, and newborn child. Glob Health Action.

[6] Bhutta ZA, Lassi ZS, Blanc A, Donnay F (2010). Linkages among reproductive health, maternal health, and perinatal outcomes. Semin Perinatol.

[7] Timmermans S, Jaddoe VW, Hofman A, Steegers-Theunissen RP, Steegers EA (2009). Periconception folic acid supplementation, fetal growth and the risks of low birth weight and preterm birth: the Generation R Study. Br J Nutr.

[8] Bell AW, McBride BW, Slepetis R, Early RJ, Currie WB (1989). Chronic heat stress and prenatal development in sheep: I. Conceptus growth and maternal plasma hormones and metabolites. J Anim Sci.

[9] Ngo NS, Horton RM (2016). Climate change and fetal health: the impacts of exposure to extreme temperatures in New York City. Environ Res.

[10] Poursafa P, Kelishadi R (2011). What health professionals should know about the health effects of air pollution and climate change on children and pregnant mothers. Iran J Nurs Midwifery Res.

[11] Kjellstrom T. Climate change, direct heat exposure, health and well-being in low and middle-income countries. Glob Health Action. 2009;2. doi:10.3402/gha.v2i0.1958. PubMed PMID: 20027264; PubMed Central PMCID: PMC2780846. 10.3402/gha.v2i0.1958 PMC2780846 20027264

[12] Sharma R, Biedenharn KR, Fedor JM, Agarwal A (2013). Lifestyle factors and reproductive health: taking control of your fertility. Reprod Biol Endocrinol.

[13] Lopez-Gatius F, Hunter RH, Garbayo JM (2007). Plasma concentrations of pregnancy-associated glycoprotein-1 (PAG-1) in high producing dairy cows suffering early fetal loss during the warm season. Theriogenology.

[14] Mahon P, Harvey N, Crozier S (2010). Low maternal vitamin D status and fetal bone development: cohort study. J Bone Miner Res.

[15] Wohlfahrt J, Melbye M, Christens P, Andersen AM, Hjalgrim H (1998). Secular and seasonal variation of length and weight at birth. Lancet.

[16] Murray LJ, O'Reilly DP, Betts N, Patterson CC, Davey Smith G, Evans AE (2000). Season and outdoor ambient temperature: effects on birth weight. Obstet Gynecol.

[17] McGrath JJ, Keeping D, Saha S, Chant DC, Lieberman DE, O'Callaghan MJ (2005). Seasonal fluctuations in birth weight and neonatal limb length; does prenatal vitamin D influence neonatal size and shape?. Early Hum Dev.

[18] Chodick G, Shalev V, Goren I, Inskip PD (2007). Seasonality in birth weight in Israel: new evidence suggests several global patterns and different etiologies. Ann Epidemiol.

[19] Lawlor DA, Leon DA, Davey Smith G (2005). The association of ambient outdoor temperature throughout pregnancy and offspring birthweight: findings from the Aberdeen Children of the 1950s cohort. BJOG.

[20] UNICEF, The state of the world's children 2009. Maternal and newborn health. New York, NY: UNICEF; 2009.

[21] Yasmin S, Osrin D, Paul E, Costello A (2001). Neonatal mortality of low-birth-weight infants in Bangladesh. Bull World Health Organ.

[22] Villar J, Belizán JM (1982). The relative contribution of prematurity and fetal growth retardation to low birth weight in developing and developed societies. Am J Obstet Gynecol.

[23] Persson LA, Arifeen S, Ekstrom EC (2012). Effects of prenatal micronutrient and early food supplementation on maternal hemoglobin, birth weight, and infant mortality among children in Bangladesh: the MINIMat randomized trial. JAMA.

[24] Moore SE, Fulford AJ, Streatfield PK, Persson LA, Prentice AM (2004). Comparative analysis of patterns of survival by season of birth in rural Bangladeshi and Gambian populations. Int J Epidemiol.

[25] Waldie KE, Poulton R, Kirk IJ, Silva PA (2000). The effects of pre- and post-natal sunlight exposure on human growth: evidence from the Southern Hemisphere. Early Hum Dev.

[26] Langer-Gould A, Huang S, Van Den Eeden SK (2011). Vitamin D, pregnancy, breastfeeding, and postpartum multiple sclerosis relapses. Arch Neurol.

[27] Holick MF (1996). Vitamin D, and bone health. J Nutr.

[28] Macdonald HM, Mavroeidi A, Fraser WD (2011). Sunlight and dietary contributions to the seasonal vitamin D status of cohorts of healthy postmenopausal women living at northerly latitudes: a major cause for concern?. Osteoporos Int.

[29] Morley R, Carlin JB, Pasco JA, Wark JD (2006). Maternal 25-hydroxyvitamin D and parathyroid hormone concentrations and offspring birth size. J Clin Endocrinol Metab.

[30] Pereira G, Cook A, Haggar F, Bower C, Nassar N (2012). Seasonal variation in fetal growth: accounting for sociodemographic, biological, and environmental exposures. Am J Obstet Gynecol.

[31] Roy DK, Berry JL, Pye SR (2007). Vitamin D status and bone mass in UK South Asian women. Bone.

[32] Wells JC (2002). Thermal environment and human birth weight. J Theor Biol.

[33] Flouris AD, Spiropoulos Y, Sakellariou GJ, Koutedakis Y (2009). Effect of seasonal programming on fetal development and longevity: links with environmental temperature. Am J Hum Biol.

[34] Thame M, Osmond C, Wilks R, Bennett FI, Forrester TE (2001). Second-trimester placental volume and infant size at birth. Obstet Gynecol.

[35] Collier RJ, Doelger SG, Head HH, Thatcher WW, Wilcox CJ (1982). Effects of heat stress during pregnancy on maternal hormone concentrations, calf birth weight and postpartum milk yield of Holstein cows. J Anim Sci.

[36] Block J, Drost M, Monson RL (2003). Use of insulin-like growth factor-I during embryo culture and treatment of recipients with gonadotropin-releasing hormone to increase pregnancy rates following the transfer of in vitro-produced embryos to heat-stressed, lactating cows. J Anim Sci.

[37] Keller CF (2007). Global warming 2007. An update to global warming: the balance of evidence and its policy implications. Sci World J.

[38] Barker DJ, Osmond C, Kajantie E, Eriksson JG (2009). Growth and chronic disease: findings in the Helsinki Birth Cohort. Ann Hum Biol.

[39] Hille ET, Weisglas-Kuperus N, van Goudoever JB (2007). Functional outcomes and participation in young adulthood for very preterm and very low birth weight infants: the Dutch Project on Preterm and Small for Gestational Age Infants at 19 years of age. Pediatrics.

[40] Khan AI (2013). Effects of pre- and postnatal nutrition interventions on child growth and body composition: the MINIMat trial in rural Bangladesh. Glob Health Action.

