# Supplementary material for: Temperature during pregnancy influences the fetal growth and birth size
Source: Trop Med Health. 2016 Dec 14;45:1. doi: 10.1186/s41182-016-0041-6 (PMC5223368; doi:10.1186/s41182-016-0041-6)
Supplement: Additional file 1: Table S1. — Regression coefficients for the association between birth length and temperature at different gestational points (using 1-week moving average temperature; n = 3267). (DOC 34 kb) [file 41182_2016_41_MOESM1_ESM.doc]

Additional file 1: Table S1. Regression coefficients for the association between birth length and temperature at different gestational points (using 1-week moving average temperature; n=3267)

| Weeks | Multivariate* | | Multivariate** | |
| --- | --- | --- | --- | --- |
|  | *β* | P value | *β* | P value |
| 8 | -0.022 | 0.055 | -0.011 | 0.361 |
| 12 | -0.008 | 0.538 | -0.003 | 0.809 |
| 19 | 0.-007 | 0.490 | -0.010 | 0.343 |
| 24 | -0.003 | 0.743 | -0.014 | 0.142 |
| 28 | 0.003 | 0.745 | -0.007 | 0.496 |
| 30 | 0.011 | 0.209 | 0.002 | 0.973 |
| Before birth |  |  |  |  |
| 6 | 0.015 | 0.082 | 0.007 | 0.432 |
| 4 | 0.025 | 0.005 | 0.021 | 0.026 |
| 2 | 0.039 | <0.001 | 0.040 | <0.001 |
| At birth | 0.050 | <0.001 | 0.046 | <0.001 |

*Adjusted for sex of infant, BMI, mother’s education, parity, season at birth, and gestational week at birth.

**Adjusted for precipitation, sex of infant, BMI, mother’s education, parity, season at birth, and gestational week at birth.
