# Supplementary material for: Temperature during pregnancy influences the fetal growth and birth size
Source: Trop Med Health. 2016 Dec 14;45:1. doi: 10.1186/s41182-016-0041-6 (PMC5223368; doi:10.1186/s41182-016-0041-6)
Supplement: Additional file 2: Table S2. — Regression coefficients for the association between birth weight and temperature at different gestational points (using 1-week moving average temperature; n = 3267). (DOC 1478 kb) [file 41182_2016_41_MOESM2_ESM.doc]

Additional file 2: Table S2 Regression coefficients for the association between birth weight and temperature at different gestational points (using 1-week moving average temperature; n=3267)

| Weeks  8 | Multivariate* | | Multivariate ** | |
| --- | --- | --- | --- | --- |
|  | *β* | P value | *β* | P value |
| 8 | 0-517 | 0.812 | -0.758 | 0.744 |
| 12 | 5.98 | 0.020 | 6.629 | 0.011 |
| 19 | 3.058 | 0.102 | 3.781 | 0.055 |
| 24 | 0.265 | 0.876 | 0-.607 | 0.750 |
| 28 | -1.112 | 0.502 | -2.15 | 0.268 |
| 30 | 0-.966 | 0.550 | -1.151 | 0.538 |
| Before birth |  |  |  |  |
| 6 | -1.320 | 0.420 | -2.86 | 0.117 |
| 4 | -0.603 | 0.824 | 0.480 | 0.795 |
| 2 | 0.468 | 0.801 | 1.627 | 0.409 |
| At birth | 1.310 | 0.525 | 1.385 | 0.517 |

*Adjusted for sex of infant, BMI, mother’s education, parity, season, and gestational week at birth.

**Adjusted for precipitation, sex of infant, BMI, mother’s education, parity, season, and gestational week at birth.
