# Supplementary material for: Temperature during pregnancy influences the fetal growth and birth size
Source: Trop Med Health. 2016 Dec 14;45:1. doi: 10.1186/s41182-016-0041-6 (PMC5223368; doi:10.1186/s41182-016-0041-6)
Supplement: Additional file 3: Table S3. — a. Variance inflation factor (VIF) of birth length at 6 weeks before birth. b. Variance inflation factors (VIF) of birth length at 4 weeks before birth. c. Variance inflation factors (VIF) of birth length at 2 weeks before birth. d. Variance inflation factors (VIF) of birth length at birth. (DOC 1119 kb) [file 41182_2016_41_MOESM3_ESM.doc]

Additional file 3: Table S3a. Variance inflation factor (VIF) of birth length at 6 weeks before birth

| Model* | | Model** | | Model*** | |
| --- | --- | --- | --- | --- | --- |
| Variables | VIF | Variables | VIF | Variables | VIF |
| GA at birth | 1.00 | GA at birth | 1.01 | GA at birth | 1.01 |
| Season | 1.13 | Seasons | 1.13 | Seasons | 1.40 |
|  |  | Infant sex | 1.00 | Infant sex | 1.00 |
|  |  | parity | 1.00 | parity | 1.00 |
|  |  | education | 1.01 | Education | 1.01 |
|  |  | BMI | 1.01 | BMI | 1.01 |
|  |  |  |  | Precipitation | 1.73 |

Table S3b. Variance inflation factors (VIF) of birth length at 4 weeks before birth

| Model* | | Model** | | Model*** | |
| --- | --- | --- | --- | --- | --- |
| Variables | VIF | Variables | VIF | Variables | VIF |
| GA at birth | 1.00 | GA at birth | 1.01 | GA at birth | 1.01 |
| Season | 1.28 | Seasons | 1.28 | Seasons | 1.76 |
|  |  | Infant sex | 1.00 | Infant sex | 1.00 |
|  |  | parity | 1.00 | parity | 1.00 |
|  |  | education | 1.01 | Education | 1.01 |
|  |  | BMI | 1.01 | BMI | 1.01 |
|  |  |  |  | Precipitation | 1.92 |

Table S3c. Variance inflation factors (VIF) of birth length at 2 weeks before birth

| Model* | | Model** | | Model*** | |
| --- | --- | --- | --- | --- | --- |
| Variables | VIF | Variables | VIF | Variables | VIF |
| GA at birth | 1.00 | GA at birth | 1.01 | GA at birth | 1.01 |
| Season | 1.51 | Seasons | 1.51 | Seasons | 1.86 |
|  |  | Infant sex | 1.00 | Infant sex | 1.00 |
|  |  | parity | 1.00 | parity | 1.00 |
|  |  | education | 1.01 | Education | 1.01 |
|  |  | BMI | 1.01 | BMI | 1.01 |
|  |  |  |  | Precipitation | 1.69 |

Table S3d. Variance inflation factors (VIF) of birth length at birth

| Model* | | Model** | | Model*** | |
| --- | --- | --- | --- | --- | --- |
| Variables | VIF | Variables | VIF | Variables | VIF |
| GA at birth | 1.00 | GA at birth | 1.01 | GA at birth | 1.01 |
| Season | 1.52 | Seasons | 1.52 | Seasons | 1.69 |
|  |  | Infant sex | 1.00 | Infant sex | 1.00 |
|  |  | parity | 1.00 | parity | 1.00 |
|  |  | education | 1.01 | Education | 1.01 |
|  |  | BMI | 1.01 | BMI | 1.01 |
|  |  |  |  | Precipitation | 1.51 |

*Adjusted for sex of infant, BMI, mother’s education, parity, season at birth, and gestational week at birth.

**Adjusted for precipitation, sex of infant, BMI, mother’s education, parity, season at birth, and gestational week at birth.
